# Location-Dependent Human Osteoarthritis Cartilage Response to Realistic Cyclic Loading: *Ex-Vivo* Analysis on Different Knee Compartments

**DOI:** 10.3389/fbioe.2022.862254

**Published:** 2022-06-15

**Authors:** Elisa Assirelli, Paolo Caravaggi, Antonio Mazzotti, Francesco Ursini, Alberto Leardini, Claudio Belvedere, Simona Neri

**Affiliations:** ^1^ Laboratory of Immunorheumatology and Tissue Regeneration, IRCCS Istituto Ortopedico Rizzoli, Bologna, Italy; ^2^ Laboratory of Movement Analysis and Functional Evaluation of Prosthesis, IRCCS Istituto Ortopedico Rizzoli, Bologna, Italy; ^3^ I Orthopaedic and Traumatologic Clinic, IRCCS Istituto Ortopedico Rizzoli, Bologna, Italy; ^4^ Rheumatology Unit, IRCCS Istituto Ortopedico Rizzoli, Bologna, Italy; ^5^ Department of Biomedical and Neuromotor Science, IRCCS Istituto Ortopedico Rizzoli, University of Bologna, Bologna, Italy

**Keywords:** osteoarthritis, knee cartilage, knee biomechanics, knee loading, knee compartments

## Abstract

**Objective:** Osteoarthritis (OA) is a multifactorial musculoskeletal disorder affecting mostly weight-bearing joints. Chondrocyte response to load is modulated by inflammatory mediators and factors involved in extracellular cartilage matrix (ECM) maintenance, but regulatory mechanisms are not fully clarified yet. By using a recently proposed experimental model combining biomechanical data with cartilage molecular information, basally and following *ex-vivo* load application, we aimed at improving the understanding of human cartilage response to cyclic mechanical compressive stimuli by including cartilage original anatomical position and OA degree as independent factors.

**Methods:** 19 mono-compartmental Knee OA patients undergoing total knee replacement were recruited. Cartilage explants from four different femoral condyles zones and with different degeneration levels were collected. The response of cartilage samples, pooled according to OA score and anatomical position was tested *ex-vivo* in a bioreactor. Mechanical stimulation was obtained via a 3-MPa 1-Hz sinusoidal compressive load for 45-min to replicate average knee loading during normal walking. Samples were analysed for chondrocyte gene expression and ECM factor release.

**Results:** Non parametric univariate and multivariate (generalized linear mixed model) analysis was performed to evaluate the effect of compression and IL-1β stimulation in relationship to the anatomical position, local disease severity and clinical parameters with a level of significance set at 0.05. We observed an anti-inflammatory effect of compression inducing a significant downmodulation of IL-6 and IL-8 levels correlated to the anatomical regions, but not to OA score. Moreover, ADAMTS5, PIICP, COMP and CS were upregulated by compression, whereas COL-2CAV was downmodulated, all in relationship to the anatomical position and to the OA degree.

**Conclusion:** While unconfined compression testing may not be fully representative of the *in-vivo* biomechanical situation, this study demonstrates the importance to consider the original cartilage anatomical position for a reliable biomolecular analysis of knee OA metabolism following mechanical stimulation.

## 1 Introduction

Osteoarthritis (OA) is a highly prevalent multifactorial rheumatic musculoskeletal disorder affecting mostly, but not exclusively, weight-bearing joints. About 300 M people globally were reported to suffer from OA in 2017 (Spencer et al., 2018). Its incidence increases with age (Spencer et al., 2018) and is the most common cause of pain and limitations of activities, resulting in considerable healthcare economic burden. In the worst cases, partial- or total-joint replacements are necessary for pain relief and to restore the original joint function. These are complex and expansive surgical procedures, whose medium and long-term outcome are not always satisfactory (Klem et al., 2020) (Hadi et al., 2016).

OA is characterized by a dysregulation of cartilage extracellular matrix (ECM) homeostasis (Guilak et al., 2018) and clinical trials and animal studies have provided strong evidence that mechanical factors can trigger OA onset and contribute to the imbalance of cartilage metabolism. The mechanical loading environment (e.g. magnitude, frequency, and distribution of loads) is a critical factor to understand OA pathophysiology and to improve treatments (Andriacchi et al., 2004). In the knee joint it is hypothesized that the combination of rolling and sliding motion (i.e. medial-pivoting motion (Zhou et al., 2021)), along with the large tibio-femoral compressive forces (Kutzner et al., 2010; Qi et al., 2013), can damage the knee cartilage and initiate OA. Abnormal weight-bearing lower-limb mechanical axis, resulting in excessive knee varism or valgism, and altered knee joint kinematics are widely known worsening factors which can result in severe knee OA (Moschella et al., 2006). Varus knee, in particular, has been reported to be more prone to medial knee compartment injury and degeneration (Sharma et al., 2010).

In terms of conservative treatments, considerable interest is being shown to the role of physical activity to prevent and slow down OA onset and progression to delay, as long as possible, the surgical intervention. The importance of physical exercise in preserving joints health in terms of cartilage biology and pathophysiology has been widely reported. High-intensity training, such as competitive sports, or sedentary behaviours with low levels of activity are associated to cartilage loss and to OA onset and progression (Leong et al., 2011; Antony et al., 2015). On the other hand, moderate exercise was shown to be able to reduce pain in knee OA (Oakeshott et al., 1988; Knapik et al., 2018), in accordance to the observation that physiologic loading contributes to tissue homeostasis and stimulates tissue anabolism (Martínez-Moreno et al., 2019).

Strong links between inflammation, mechanical load and cartilage homeostasis have been reported (Leong et al., 2011). It is understood that inflammation mediators, as well as factors involved in maintaining the extracellular cartilage matrix, play a significant role in modulating the response of chondrocytes to mechanical load (Sanchez-Adams et al., 2014). While the importance of these factors on OA pathogenesis and progression is widely recognized, the regulatory mechanisms are not fully clarified yet, and few studies on human chondrocytes, particularly on native human cartilage tissue cultures, are currently available. In a previous study by the same authors (Dolzani et al., 2019) it was found that physiological compression of human OA cartilage modulates the inflammatory milieu by differently affecting the expression of components and homeostasis regulators of the cartilage extracellular matrix. Recently, we have proposed an integrated experimental model combining biomechanical data with molecular information on tissue homeostasis, basally and following the application of *ex-vivo* loading, with a specific proof of concept application corresponding to normal walking activity (Caravaggi et al., 2021)^,^ (Zhang et al., 2020). In fact, cartilage response to loading is largely dependent on the mechanical stimuli and on the structural integrity of the tissue (Chen et al., 2013).

In this study, we aimed at improving the understanding of human cartilage response to cyclic mechanical compressive stimuli by taking into consideration two critical independent parameters: 1) the cartilage original anatomical position, and 2) the OA degree. In this study, we hypothesized that OA degree and anatomical position may affect cartilage response to loading.

## 2 Materials and Methods

### 2.1 Estimation of Knee Joint Loading

Gait data from a knee OA patient (male; age = 52 years; weight = 73 kg; height = 1.73 m; Body-Mass-Index (BMI) = 24.4 kg/m^2^) were used to estimate the loading conditions (Caravaggi et al., 2021). Knee joint flexion/extension angles and adduction/abduction moments (Leardini et al., 2007) were recorded in five comfortable walking-speed trials at 100 Hz and time-normalized over stance phase duration. The average knee contact force was obtained by using the linear relationship between knee adduction moment and contact forces reported by Kutzner et al. (Kutzner et al., 2013). Average knee pressure at the medial compartment was estimated by dividing the knee contact force by the contact area reported by Liu et al. (Liu et al., 2010). The estimated average peak pressure of 3 MPa, which is consistent also with what reported by Gilbert et al. (Gilbert et al., 2014) as contact stress at the knee in walking, set the maximum pressure for the sinusoidal pressure/time waveform used as loading condition for the cartilage samples (Caravaggi et al., 2021). A waveform frequency of 1 Hz was chosen to simulate a typical knee joint compression/relaxation pattern during normal walking. Each sample was subjected to cyclic compression for 45 min. This time was chosen to simulate a moderate stimulation of the knee, slightly above the minimum recommended exercise time of around 30 min per day (Lundebjerg, 2001) (Loew et al., 2012).

### 2.2 Sample Recruitment and Experimental Design

For study purposes, 19 mono-compartmental knee OA cases (Female/Male: 10/9; age±SD: 71 ± 9 years; mean BMI±SD: 27.3 ± 4.1 kg/m^2^; 13 medial compartment OA due to varus knee, six lateral compartment OA due to valgus knee) undergoing total knee replacement surgery were recruited. Femur’s mechanical axis alignments were 171.5 ± 4.8 deg and 174.6 ± 3.6 deg for varus and valgus cases, respectively. In mono-compartmental knee OA (with predominant biomechanical aetiology), the medial or lateral compartment in varus and valgus knee, respectively, is mainly degenerated compared to the contralateral one (Felson et al., 2013; Sharma et al., 2010, PMID 20511608). This allowed to compare articular compartments showing different cartilage degeneration within the same articular milieu, thus representing different stages of OA progression with minimum variability (paired samples). Accordingly, cartilage from the same knee was separated in affected areas, i.e., where tissue histology showed the worst score, and contralateral areas showing lower degeneration.

**FIGURE 1:**
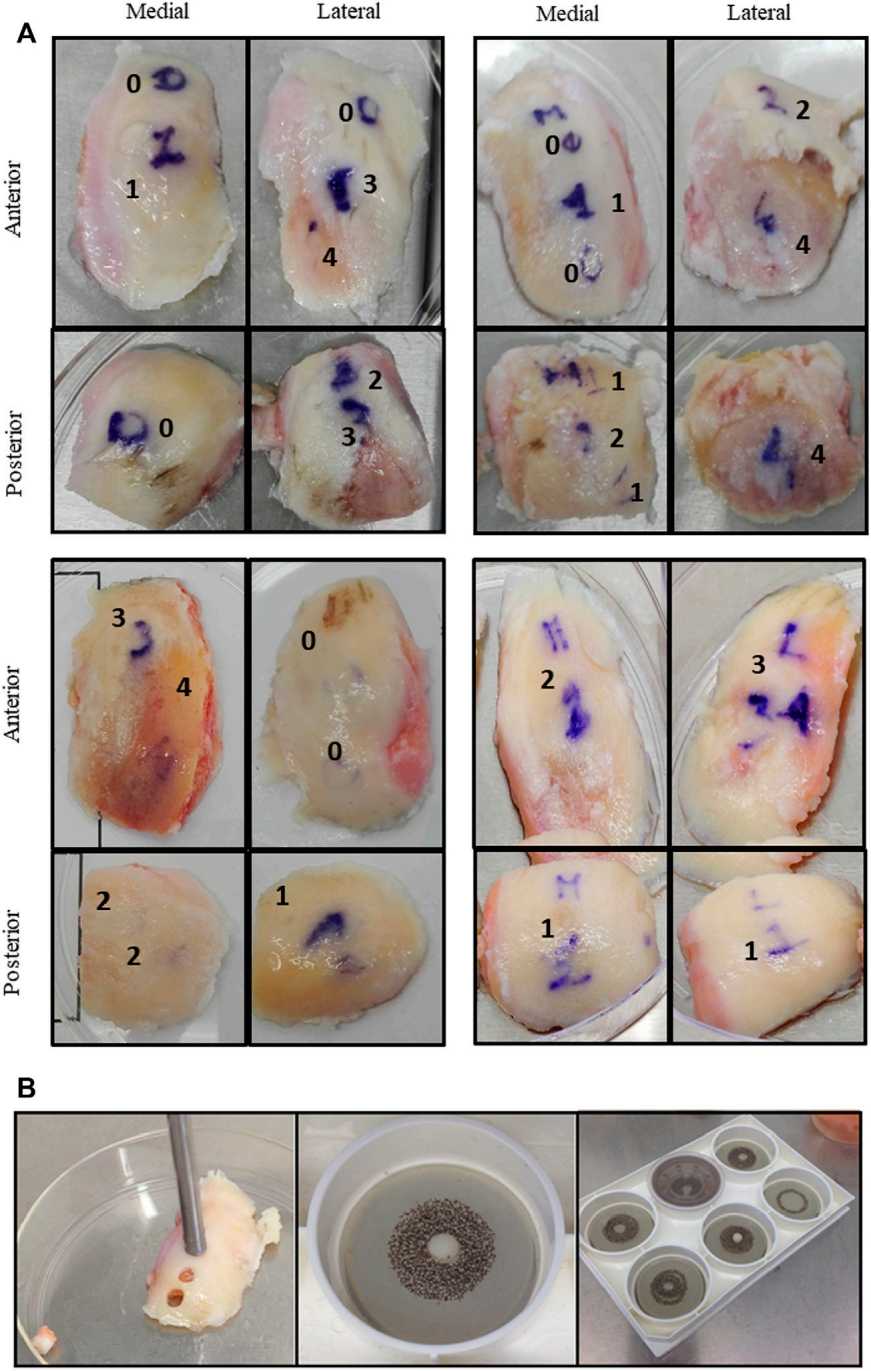
**(A)** Cartilage explants from four representative donors. Images of surgical samples are shown. Condyles were divided in zones (anterior, posterior, medial, and lateral) and in areas with different macroscopic scores (from 0 to 4). **(B)** Each area was independently cored to obtain cartilage cylinders placed inside compression plates and exposed to unconfined compression in the Flexcell bioreactor apparatus.

The study was approved by the local Ethic Committee (CE-AVEC Prot. Kneeload N. EM603-2018_89/2015/Sper/IOR_EM1) and written patient consent was recorded from all participants. Medial and lateral femoral condyles were collected at time of knee replacement surgery as described in Caravaggi et al., 2021 (Caravaggi et al., 2021). Medial and lateral condyles were divided in four topographical areas, corresponding to the original anatomical location and to different *in-vivo* loading conditions: medial anterior, medial posterior, lateral anterior and lateral posterior (Figure 1A). Differentiating the condylar region, as medial and lateral compartments, and within the same compartment, between anterior and posterior areas, is critical to apply the correct biomechanical conditions and to obtain more in-depth information on the biological modulations of cartilage samples which are consistent in terms of long-term wear and mechanical loadings *in-vivo* (Caravaggi et al., 2021).

A macroscopic score was assigned by the surgeon to all the identified areas, according to the Collins grading system of disease severity (from 0 to 4, with 0 corresponding to apparently normal cartilage, and 4 corresponding to completely degenerated tissue) (Pritzker et al., 2006; Caravaggi et al., 2021). Each area could appear homogeneous (with only one attributed macroscopic score) or further zoned in sub-areas in case of heterogeneous tissue degeneration. In the latter case, subzones from the same area were separately recovered and further analysed.

Cylinders of cartilage tissue were harvested with an 8 gauge-diameter corer (internal diameter: 2.5 mm) after subchondral bone removal and cultured overnight in D-MEM (SIGMA, Sigma Aldrich, St. Louis, United States) in serum starvation conditions. *Ex-vivo* mechanical compression was applied with the FlexCell FX-4000C stage presser apparatus (Flexcell International Corporation, United States), allowing to homogenously apply uniform loading to different specimens. Fresh cartilage cylinders were weighed and positioned into compression plate wells (consisting of a chamber bonded to the bottom of a flexible silicon rubber membrane) with 2 ml D-MEM without FCS. The cylinders were carefully oriented, all placed with the upper cartilage layer facing up. A stationary platen was added to each well and screwed down to the top of the tissue. Loading uniformity was guaranteed by the positioning of only one cartilage cylinder per well, therefore each compression chamber was set at the specific height of the sample. Air pressure applied to the bottom caused the chamber to rise and apply an unconfined compression to explants. All the experiments were performed at 37°C, 5% CO_2_. A 3 MPa amplitude 1 Hz frequency sinusoidal compressive load was applied for 45 min. Paired control explants were maintained at 37°C, 5% CO_2_ in unloaded conditions. For each donor, two conditions were tested: basal and stimulated with 2 ng/ml Interleukin (IL)-1β (pro-inflammatory stimulus; rhIL-1β, R&D Systems, Minneapolis, United States). For each experimental condition, two/three explants (depending on available cartilage) were used. After compression, cartilage samples and culture supernatants were immediately recovered and frozen (liquid nitrogen and −80°C, respectively) for molecular analyses and soluble factor determination.

A series of markers of cartilage turnover was identified to evaluate the effect of *ex vivo* loading. Some markers were quantified as mRNAs and others as proteins released in culture supernatants. Table 1 is reporting all the analyzed variables and their main function in cartilage metabolism.

**TABLE 1:** Cartilage markers analysed for their gene expression or as soluble factors released in culture supernatants. The main function of each marker in cartilage turnover is reported.

Markers	Description/role in cartilage biology	References
COL2A1	One of the main components of cartilage extracellular matrix, it is fundamental for cartilage structure maintenance	Guilak et al. (2018)
SOX9	A transcription factor that regulates the expression of genes including COLIIA1 and ACAN, respectively encoding for COL2A1 and Aggrecan	(Liu et al., 2017) (Song and Park, 2020)
Aggrecan	An extracellular matrix component, it is the most crucial proteoglycan for proper functioning of articular cartilage	Guilak et al. (2018)
IL-4Rα	IL-4 is an antinflammatory cytokine with a known protective anabolic effect on articular cartilage. It is also involved in mechanotransduction. In osteoarthritic chondrocytes an alteration of the IL-4/IL-4R system is described	Silvestri et al. (2006)
C3 CFB	This two factors, members of the Complement system alternative pathway, contribute to activation of the innate immunity response in OA joints	(Haseeb and Haqqi, 2013) (Assirelli et al., 2020)
IL-6	It is a cytokine that strongly activates the immune system and enhances the inflammatory response. Its production in the affected joint is usually in response to IL-1β and TNFα and is mainly implemented by chondrocytes, osteoblasts, fibroblast-like synoviocytes, macrophages, and adipocytes. IL-6 on joint cartilage causes a decrease in the production of type II collagen and increases the production of enzymes from the metalloproteinase group	Wojdasiewicz et al. (2014)
IL-8	This chemokine is an important inflammatory mediator that plays a central role in the onset of inflammatory processes in OA. Increased levels of IL-8 in synovial fluid are associated with pain	Molnar et al. (2021)
MMP13	One of the main matrix enzymes involved in cartilage degradation, it is a collagenase responsible of type II collagen cleavage, and it plays a crucial role in OA pathogenesis and progression	(Mehana et al., 2019) (Hu and Ecker, 2021)
ADAMTS5	One of the main matrix enzymes involved in cartilage degradation, it is an aggrecanase also involved in OA cartilage proteoglycan loss	Jiang et al. (2021)
COMP	A noncollagenous extracellular matrix protein, it is a biomarker of cartilage turnover	Bay-Jensen et al. (2016)
CS	It is an important structural component of cartilage, providing resistance to compression	Kiani et al. (2002)
PIICP	It is a product of cartilage metabolism, considered as biological marker of collagen II synthesis	Nemirovskiy et al. (2008)
CoL-2CAV	It is a product of cartilage metabolism, considered as biomarker of collagenase cleavage of collagen II	Elsaid and Chichester, (2006)

### 2.3 Microscopic Cartilage Scoring

Before compression, one cartilage sample from each sub-area was used for microscopic cartilage scoring to confirm the previously attributed macroscopic score. Samples were freshly collected, formalin fixed, and paraffin embedded until use. A representative number of cartilage samples from 16 out of 19 OA cases was scored after conventional Safranin O-staining. Briefly, 5 μm thick sections of FFPE samples were rehydrated and stained with Hematoxylin/Eosin, 0.1% Safranin-O and 0.02% Fast Green (SIGMA-ALDRICH, Munich, Germany). One slice from each sub-area was independently analyzed by two experienced biologists for histopathology grading score attribution following the OARSI criteria (Pritzker et al., 2006). Scores attributed ranged from 0 (intact cartilage), to 4 (complete cartilage degradation). Grades 5 and 6 were never attributed since the osteochondral bone was excluded from the analysis. The entire section was evaluated, and the analysis performed with an Eclipse 90i microscope and NIS elements software (NIKON CORPORATION, Tokyo, Japan).

### 2.4 Gene Expression Analysis

Depending on the available material, one or two cartilage cylinders (20–80 mg) for each experimental condition (and for each donor sample) were dedicated to gene expression analysis, as described in Caravaggi et al., 2021 (Caravaggi et al., 2021). Briefly, liquid nitrogen frozen samples were pulverized using the Mikro-Dismembrator S (Sartorius Stedim Italy SpA, Italy) grinding mill in 5 ml PFTE shaking flasks with a stainless-steel grinding ball at a shaking frequency of 2000/min, for 45 s. Total cellular RNA was extracted using RNApure (EUROCLONE, Milan, Italy) and reverse transcribed using SuperScript VILO cDNA Synthesis kit (INVITROGEN, Life Technologies, NY, United States) by random hexamer priming, following manufacturer’s instructions.

Messenger RNA expression was evaluated by semi-quantitative Real-Time RT-PCR in a Light Cycler Instrument (ROCHE Molecular Biochemicals, Mannheim, Germany) using the SYBR Premix Ex Taq (TAKARA Biomedicals, Tokyo, Japan) with the following protocol: 95°C for 10 s, 45 cycles at 95°C for 5 s and 60°C for 20 s. Analyzed genes and primers sequences are shown in Supplementary Table S1.

Cycle Threshold (C_T_) values were determined for each sample. Specificity of the amplicons was checked by gel electrophoresis and confirmed at each run by melting curve analysis. Amplification efficiency (above 90% for each primer couple) was evaluated using 10-fold serial dilutions of positive control cDNAs and calculated from the slopes of log input amounts plotted versus crossing point values. Expression levels were quantified in respect to the glyceraldehyde-3-phosphate dehydrogenase (GAPDH) housekeeping gene following formula (1 + E)^ΔC^_T_, where E represents the reaction efficiency and ΔC_T_ the difference between the GAPDH crossing point and the specific crossing point for each sample.

### 2.5 Soluble Factor Quantification

Matrix MetalloProteinase 13 (MMP13), a Disintegrin And Metalloproteinase with Thrombospondin motif 5 or Aggrecanase-2 (ADAMTS-5), Aggrecan (Aggr), Cartilage Oligomeric Matrix Protein (COMP), Procollagen II C-Terminal Propeptide (PIICP), Chondroitin Sulfate (CS), and Collagen Type 2 Cleavage (COL-2CAV) were measured by ELISA in culture supernatants recovered after compression, according to manufacturer’s instructions (Cloud-Clone Corp, United States for MMP13, ADAMTS-5, Aggr, COMP, PIICP and CS kits; MyBioSource, United States for COL-2CAV) using Infinite^®^ M Plex multimode microplate reader equipped with Magellan Software (Tecan Trading AG, Switzerland) and a programmable microplate washer (Hydro flex Tecan Trading AG, Switzerland). Results were normalized to mg of tissue of the corresponding explant. Due to bioreactor technical requirements, each cartilage cylinder was cultured in 2 ml medium, resulting in high dilution of released factors in supernatants. For this reason, supernatants were vacuum concentrated before ELISA test.

### 2.6 Statistical Analysis

Data are presented as medians, 25^th^ and 75^th^ percentiles, minimum and maximum values; percentages, means ± standard deviations, as appropriate.

The main analysis was the Generalized Linear Mixed Models (GLMM) analysis with cartilage markers as dependent variables; articular tissue sample position and grading, knee OA compartment, IL-1β stimulus and compression as fixed effects. The analysis performed resulted in estimated means with 95% CI; the comparisons between the subgroups were performed using the Sidak multiple comparisons test. Results in graphs and tables are resumed with medians and 25^th^–75^th^ percentiles to facilitate reading of data that were highly asymmetrical.

A flowchart of the statistical analysis is presented in Table 2. Briefly, to find the suitable distribution and link function for the model, the Shapiro test was used to assess normality of the distributions of the cartilage markers, which were all not normally distributed. Subsequently, logarithmic transformation was applied only to those markers with all positive values and the Shapiro test was used to assess log-normality of these latter markers (stage 1). If cartilage markers presented zero-values, Tweedie distribution with log-link function was used in the model; in case of absence of zero-values and not log-normal distribution the gamma distribution with log-link function was used (stage 3).

**TABLE 2:** Flowchart of the applied statistical methodology. Main analysis: Generalized Linear Mixed Model (GLMM) with cartilage markers (mRNAs and soluble factors) as Dependent Variables; compression (Compressed-Non Compressed), sample position (anterior-posterior; medial-lateral), compartment (varus-valgus), disease grading (0–1/3–4), ILβ-no ILβ, affected-contralateral as potential Fixed Effects; age, BMI, OA-Grade (Kellgren-Lawrence) as potential covariates; patient, and gender as potential random effects.

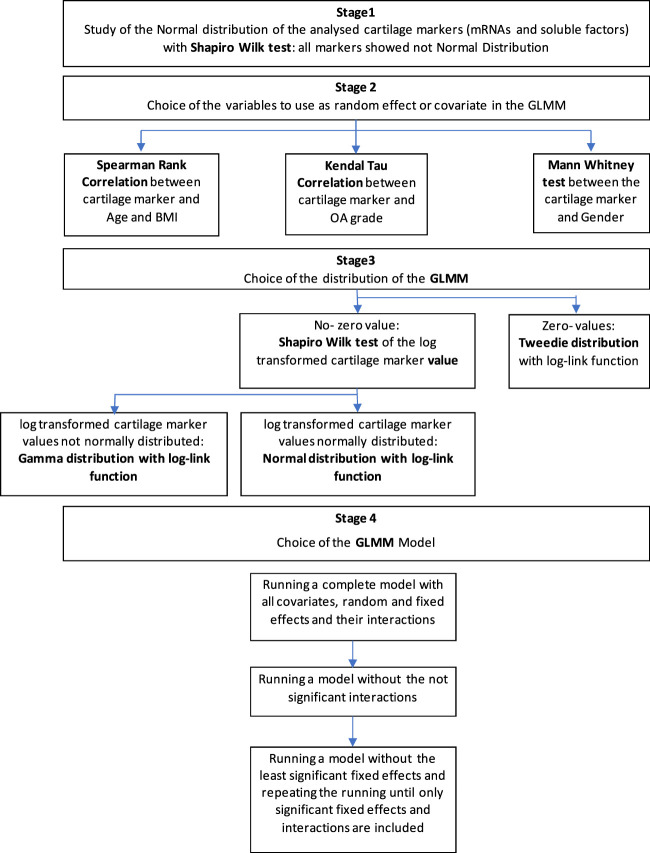

The random effects to be used in the model were chosen between age, BMI, OA grade and gender using the appropriate univariate analysis (Spearman’s Rank correlation for age and BMI; Kendall’s tau correlation OA for grade; and Mann Whitney U-test for gender) (stage 2).

To choose the right model, the complete model was firstly applied with all the interactions, and then run without the not significant interactions, possibly with only the fixed main effects (stage 4).

The Sidak test was then used to compare the results of fixed effects.

The level of statistical significance was set at *p* < 0.05. All statistical analysis was performed using SPSS v.19.0 (IBM Corp, Armonk, NY, United States).

## 3 Results

### 3.1 Patients and Samples Characterization

In Figure 1, representative examples of surgical samples with attribution of macroscopic score, together with explant collection via tissue coring, are shown. Analyses were performed separately in these areas. Patients’ characteristics are summarized in Table 3.

**TABLE 3:** Mono-compartmental knee OA patient characteristics: age at surgery (in years), sex, body mass index (BMI, in kg/m2), radiographic score (Kellgren and Lawrence, 1957) and affected compartment (medial, M, for varus, and lateral L, for valgus knees).

Age	Sex	BMI	KL	Affected compartment
78	F	22.4	3	M
67	M	28.4	3	M
76	F	25.2	3	M
75	M	24.2	2	M
79	F	—	4	L
64	M	26.9	3	M
69	F	32.9	2	M
80	F	26.9	3	L
68	M	32	3	M
82	M	28.3	4	M
72	F	29.1	3	M
66	F	37.7	2	L
87	F	26.5	3	L
66	M	24.4	3	M
80	M	26.6	3	L
53	F	20.1	3	L
56	F	22.9	2	L
61	M	28.2	4	M
70	F	28.4	3	M

**FIGURE 2:**
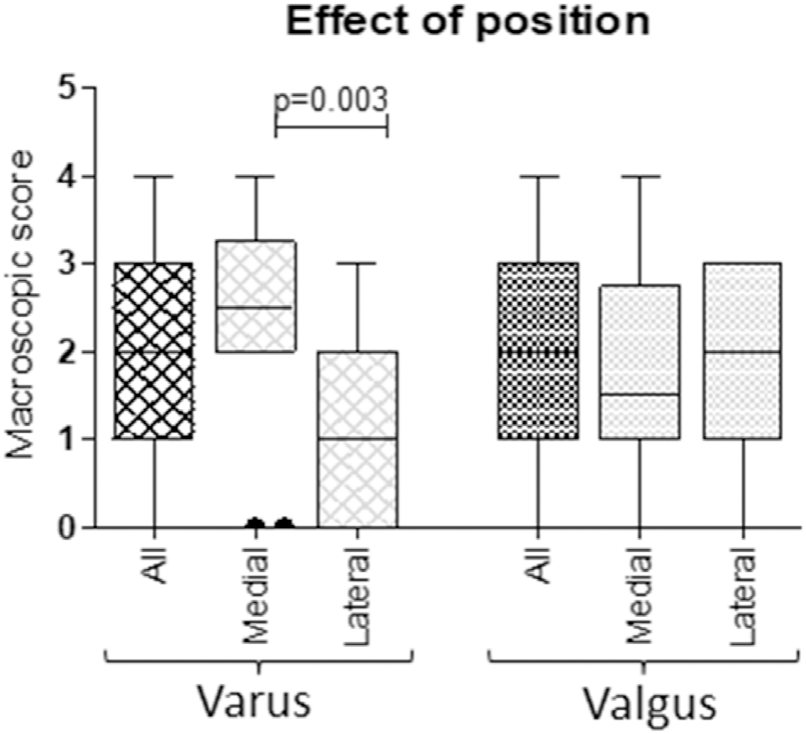
Correspondence between macroscopic scores of medial and lateral condylar compartments and varus/valgus condition. According to knee biomechanics, higher scores in medial compartments of varus knees and higher scores in lateral compartments of valgus knees were observed (Mann Whitney U test).

Samples from mono-compartmental knee OA cases, corresponding to varus (medial condylar compartment prevalently affected) or valgus knee (lateral condylar compartment prevalently affected) allowed to compare, in the same donor sample, the behaviour of areas with distinct pathology grade. Accordingly, a correspondence between the affected articular compartment and the macroscopic score was observed: varus knee cases showed higher OA scores in medial compartments (median medial value 2.50; median lateral value 1.00, *p* = 0.003), whereas valgus knee cases showed higher OA scores in lateral compartments, albeit without reaching statistical significance (median medial value 1.50; median lateral value 2.00, *p* = ns) (Figure 2).

### 3.2 Microscopic/Macroscopic Cartilage Score Correlation

**FIGURE 3:**
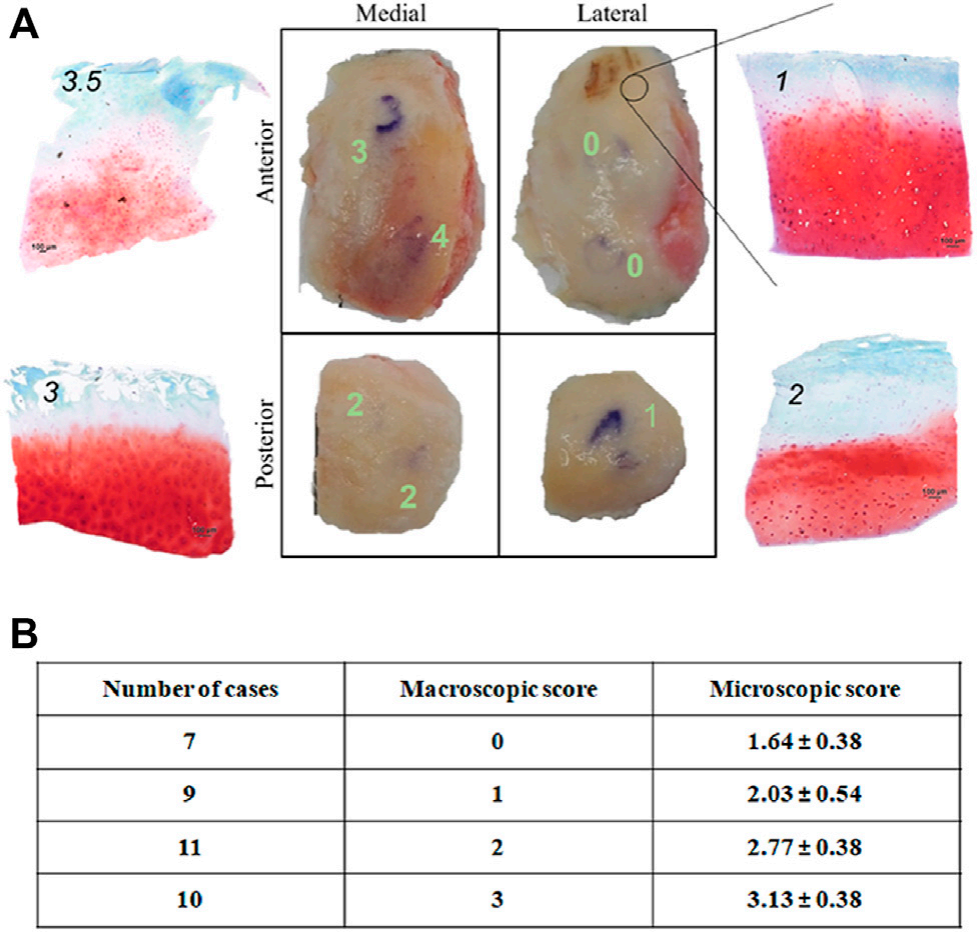
**(A)** Microscopic histological score attributio n to condyle areas identified by position (medial, lateral, anterior, posterior) and OA degree (macroscopic score) of a representative knee OA sample. Scale bar: 100 μm; **(B)** Correlation between macroscopic and microscopic (histopathological) score of 37 condyle areas from 16 donors. Microscopic scores are represented as mean ± SD.

A histopathologic score was attributed to a total of 37 previously identified cartilage areas from 16 donors. These areas carried a macroscopic score attribution from 0 to 3 (no areas with macroscopic score 4 were included in correlation analysis since this score corresponded to completely degenerated cartilage and no histology was possible). In Figure 3A, a representative example of histological evaluation in areas with different macroscopic score, is shown.

No sample (even those with macroscopic score = 0) showed histological score corresponding to 0, thus confirming that even apparently intact cartilage from an OA joint carries tissue alteration (Figure 3B). Spearman correlation analysis highlighted a positive correlation between the two scores (r = 0.99 between macroscopic scores and mean microscopic scores; Figure 3B), in accordance with previous results (Caravaggi et al., 2021). This confirms the reliability of the macroscopic score for sample partition in areas of different pathology level before compression.

### 3.3 Anatomical Position, Grading, Disease Grade and Patients’ Characteristics Influence on Cartilage Biomarkers

#### 3.3.1 Gene Expression

We firstly evaluated Aggrecan, COL2A1, and SOX9 gene expression. In general, remarkable interindividual variability was observed in basal levels of gene expression (Supplementary Table S2).

An interesting correlation (Spearman) between gene expression and BMI was observed. All the three markers correlated with BMI: inverse correlation with COL2A1 (rho = −0.224, *p* < 0.0001); direct correlation with aggrecan (rho = 0.178, *p* = 0.013) and SOX9 (rho = 0.162, *p* = 0.024). The condition of varus or valgus knee (corresponding to medial or lateral knee degeneration, respectively) also influenced gene expression: varus knees showed lower COL2A1 levels than valgus ones, this difference reaching statistical significance in COL2A1 nc IL-1 samples (COL2A1varus mean expression 65,733, COL2A1valgus mean expression 5,26,074, *p* = 0.006). This was also observed for SOX9 and Aggrecan mean levels, where varus knees showed higher levels of both biomarkers than valgus (SOX9 varus mean expression 23,488, SOX9 valgus mean expression 14,260, *p* = 0.022; Aggr varus mean expression 189,216, Aggr valgus mean expression 1,24,045, *p* = 0.016).

We also evaluated markers involved in inflammation and innate immunity. In general, no significant variations were found in relationship to anatomical position, except for CFB, whose expression was higher in medial cartilage samples (*p* = 0.007; Supplementary Table S3).

IL-8, C3, and CFB directly correlated with age (rho = 0.325 *p* = 0.014; rho = 0.268 *p* = 0.004; rho = 0.328 *p* = 0.011, respectively).

The factor mostly influencing IL-6, IL-8, and IL-4Rα expression was OA grading: all these factors were more expressed in higher grade samples. A significantly higher gene expression of IL6 (*p* = 0.016), IL-8 (*p* = 0.014), and IL4Rα (*p* = 0.036) was observed in samples with macroscopic OA score of 3-4 compared to samples with score 0–1 (Supplementary Table S3). C3 and CFB gene expression also showed a similar trend even if failing to reach statistical significance (Supplementary Table S3).

#### 3.3.2 Soluble Factor Release

MMP13 and Aggrecan amounts resulted undetectable even after supernatant concentration.

ADAMTS5 and COMP amount directly correlated with BMI (rho = 0.333, *p* = 0.011 and rho = 0.268, *p* = 0.042, respectively). Furthermore, CS production directly correlated with grading (tau = 0.405 *p* < 0.0005) and COL-2CAV production directly correlated with grading and radiographic score (tau = 0.2 *p* = 0.042 and tau = 0.241 *p* = 0.019, respectively). ADAMTS5 production was higher in varus than valgus knee (*p* = 0.027); on the contrary, COL-2CAV production was higher in valgus knee (*p* = 0.025), and in the medial compartment compared to lateral (*p* = 0.011) (Supplementary Table S4).

### 3.3 Cartilage Response to Mechanical Stimulation

Tissue response to mechanical stimulation was analyzed as the effect of compression both in relationship to the anatomical original position and to the level of cartilage degeneration. To this aim, samples from areas differently affected were compared. Based on the correlation between macroscopic score and cartilage position (Figure 2), cartilage originating from varus knee medial compartment and cartilage from valgus knee lateral compartment were considered as “affected compartment”, whereas cartilage from varus knee lateral compartment and valgus knee medial compartment were considered as “contralateral compartment”.

**FIGURE 4:**
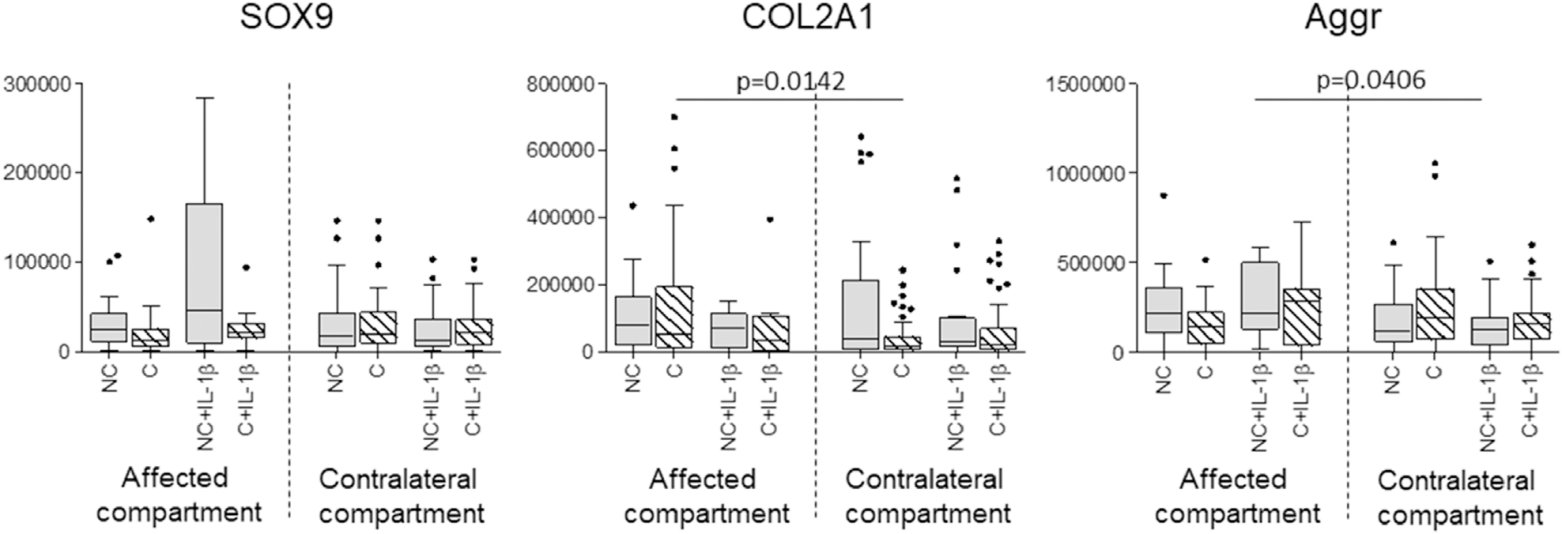
Effect of compression on SOX9, COL2A1, and Aggrecan gene expression in affected and contralateral compartments (Sidak test for multiple comparisons). Data are represented as medians (number of mRNA molecules of the gene of interest/10^5^ GAPDH copies). Boxes represent 25^th^-75^th^ percentiles; whiskers represent minimum and maximum values; dots represent outliers. NC= uncompressed; C= compressed; IL-1β= stimulated with 2 mg/ml IL-1β.

SOX9 gene expression showed no significant differences before and after compression, even in IL-1β stimulated samples; COL2A1 was reduced in contralateral compartment compressed unstimulated samples (*p* = 0.0142) compared to the affected compartment and aggrecan was reduced in uncompressed stimulated samples of the contralateral compartment compared to the affected compartment (*p* = 0.04) (Figure 4).

**FIGURE 5:**
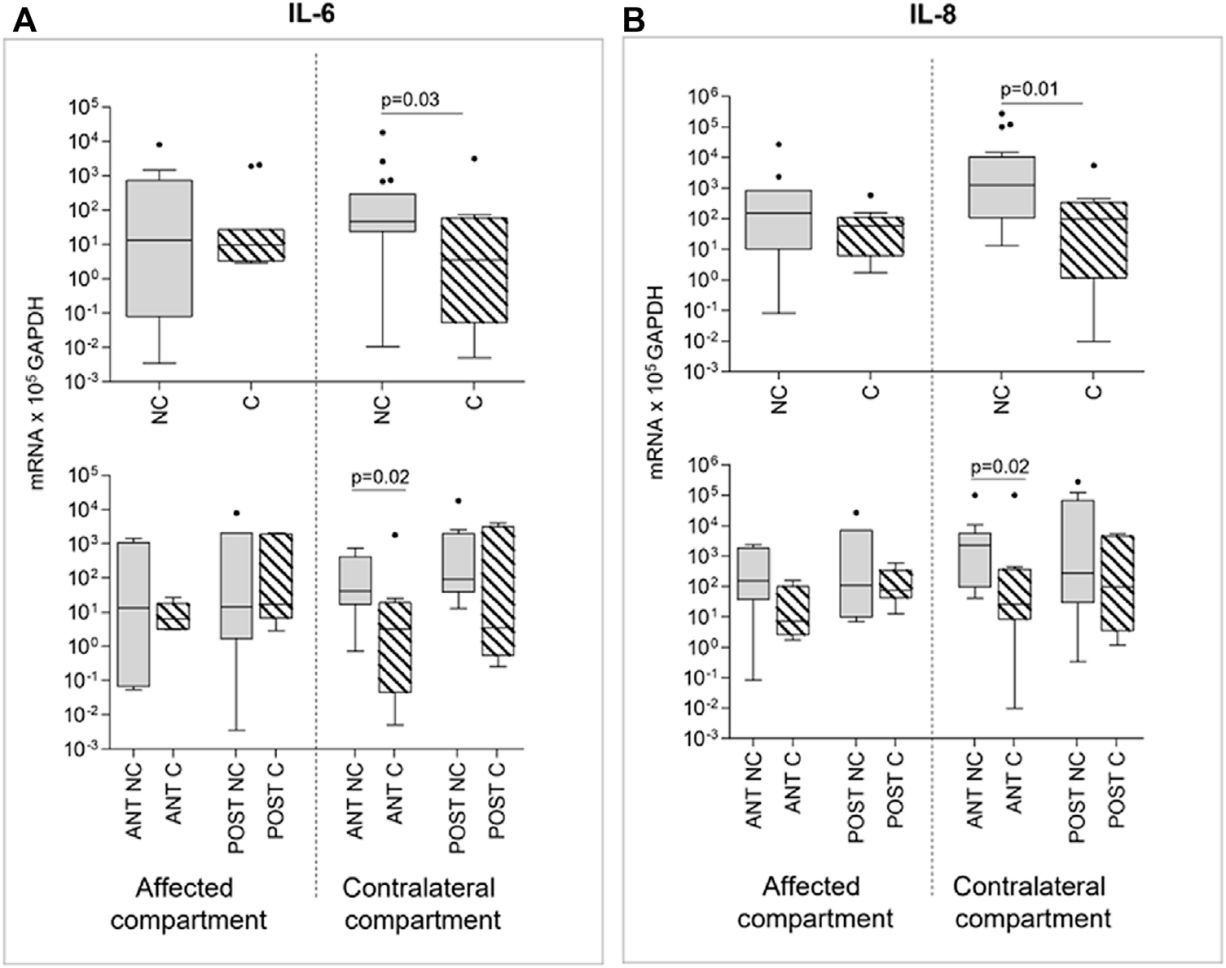
Effect of compression on IL-6 **(A)** and IL-8 **(B)** gene expression in affected and contralateral compartments (upper panels). In lower panels, samples are divided according to the original position of the cartilage area (anterior/posterior condyle) (Sidak test for multiple comparisons). Data are represented as medians (number of mRNA molecules of the gene of interest/10^5^ GAPDH copies). Boxes represent 25^th^–75^th^ percentiles; whiskers represent minimum and maximum values; dots represent outliers. NC= uncompressed; C= compressed; IL-1β= stimulated with 2 mg/ml IL-1β.

The analysis of markers involved in inflammation and innate immunity as well as soluble factors, was performed in a sub-group of IL-1β stimulated samples (n = 60). Interestingly, we observed a downmodulation of IL-6 and IL-8 expression after compression. This only happened in contralateral compartment cartilage samples (*p* = 0.0126 and *p* = 0.0327, respectively), whereas compression failed in downmodulating IL-6 and IL-8 expression in the affected compartment (Figure 5). Moreover, if considering the effect of compression in relationship to the anatomical position, a prevalent effect of compression is evident in the anterior contralateral compartments compared to posterior. In fact, the downmodulation of both IL-6 and IL-8 gene expression appears in both compartments, but it reaches statistical significance only in the anterior one (*p* = 0.02 for IL-6 and *p* = 0.02 for IL-8) (Figure 5).

**FIGURE 6:**
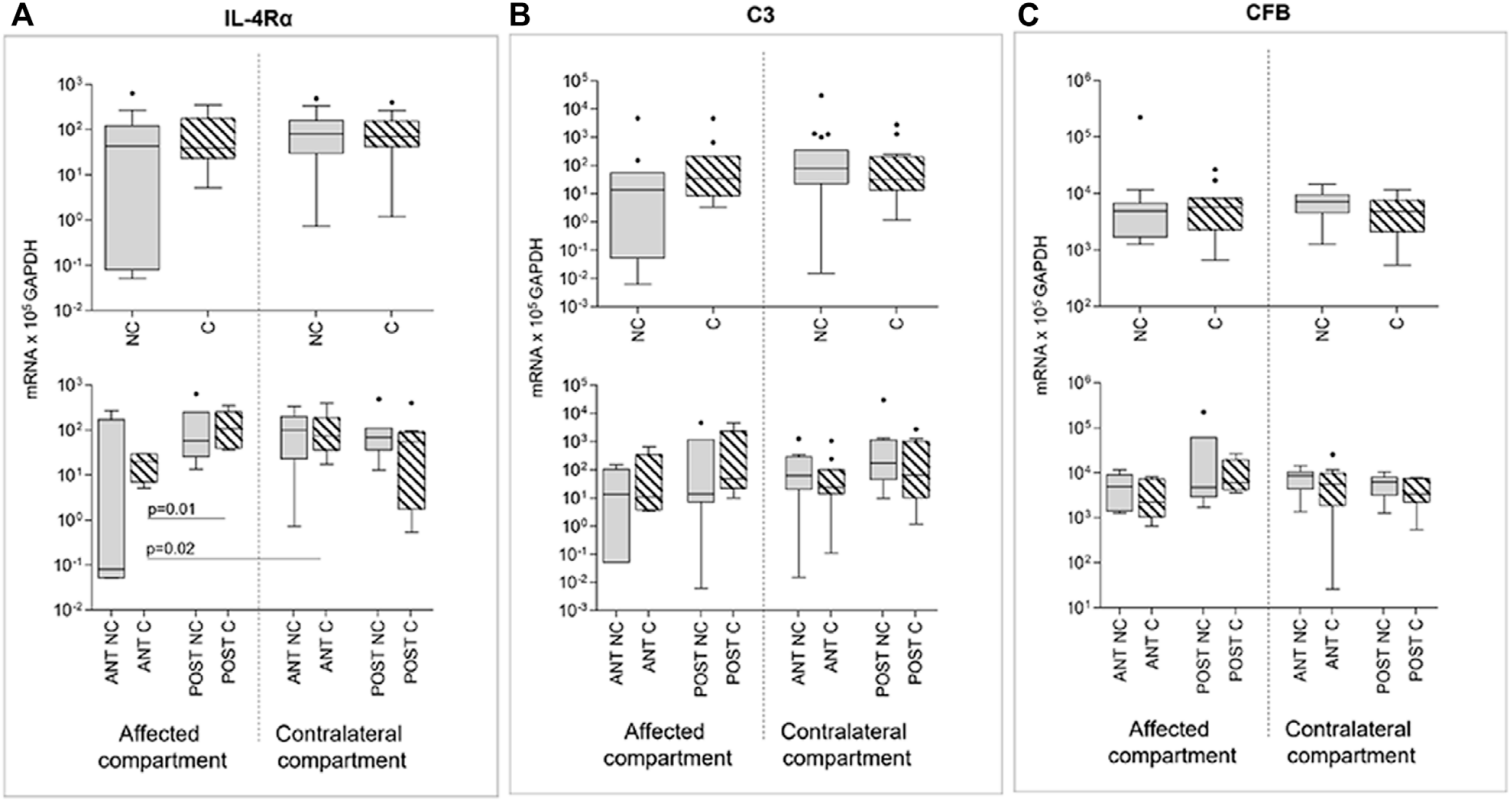
Effect of compression on IL-4Rα **(A)**, C3 **(B)**, and CFB **(C)** gene expression in affected and contralateral compartments (upper panels). In lower panels, samples are divided according to the original position of the cartilage area (anterior/posterior condyle) (Sidak test for multiple comparisons). Data are represented as medians (number of mRNA molecules of the gene of interest/10^5^ GAPDH copies). Boxes represent 25^th^–75^th^ percentiles; whiskers represent minimum and maximum values; dots represent outliers. NC, uncompressed; C, compressed; IL-1β, stimulated with 2 mg/ml IL-1β.

Compression produced no effect on IL-4Rα, C3, and CFB expression (Figure 6). Even for these markers, differences in gene expression emerged if comparing anterior and posterior areas: IL4-Rα anterior areas of the affected compartment presented, after compression, significantly lower gene expression values than posterior areas of the same compartment and of the contralateral anterior compartment, again highlighting position-dependent differences (Figure 6A).

**FIGURE 7:**
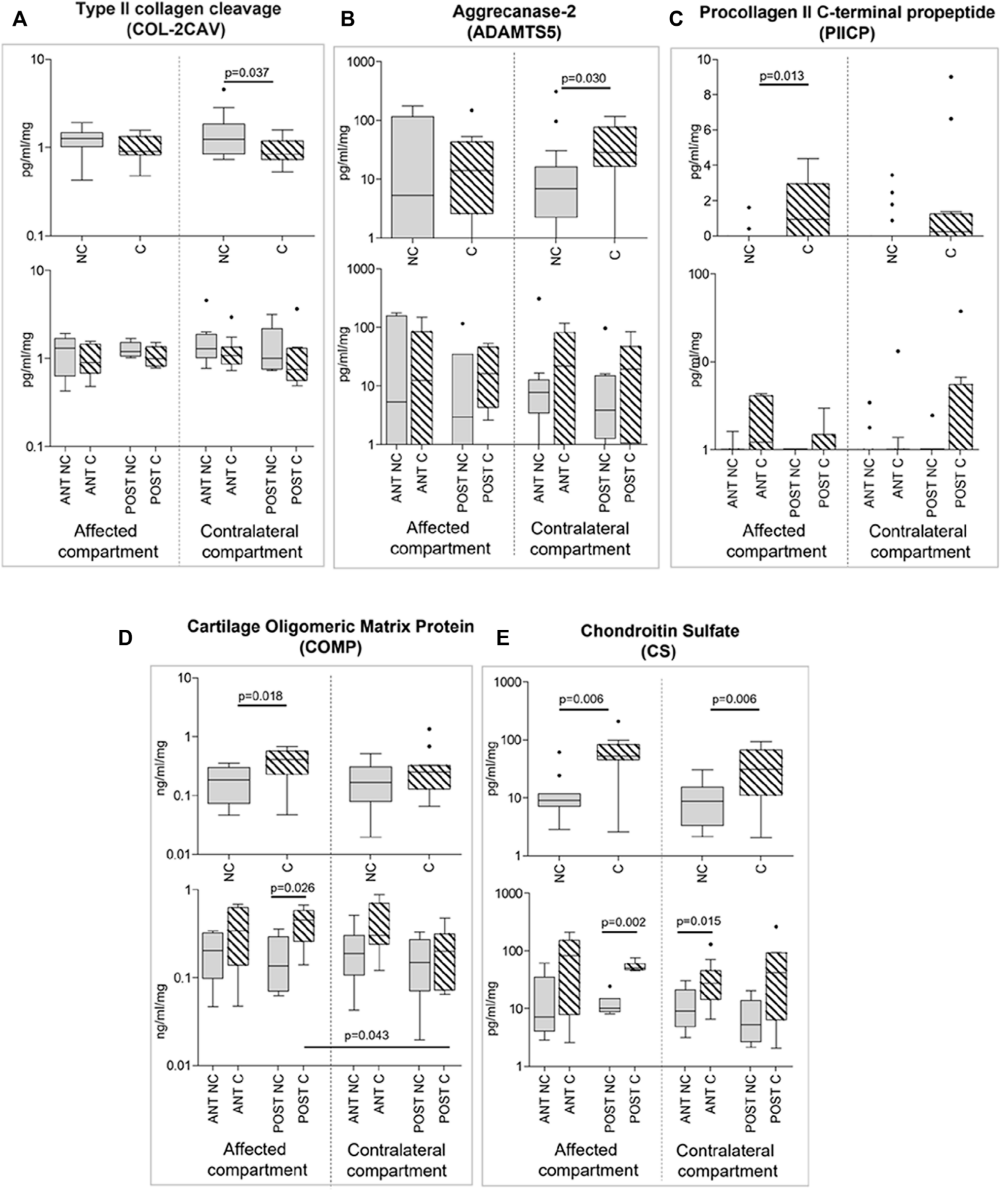
Effect of compression on soluble factor release **(A-E)** in affected and contralateral compartments (upper panels of each section). In lower panels of each section, samples are divided according to the original position of the cartilage area (anterior/posterior condyle) (Sidak test for multiple comparisons). Data are presented as medians (number of mRNA molecules of the gene of interest/10^5^ GAPDH copies). Boxes represent 25^th^– 75^th^ percentiles; whiskers represent minimum and maximum values; dots represent outliers. NC, uncompressed; C, compressed; IL-1β, stimulated with 2 mg/ml IL-1β.

We noticed that soluble factors were the most affected by compression among the molecules analyzed. Indeed, COL-2CAV release was significantly downmodulated by compression only in contralateral compartment cartilage samples (*p* = 0.0367); moreover, for this marker, anterior and posterior areas showed the same trend (Figure 7A).

ADAMTS5 expression resulted, on the contrary, upregulated by compression in the contralateral compartment (*p* = 0.030; Figure 7B); the same trend, even without reaching statistical significance, was observed in the affected compartment and both in the anterior and posterior areas (Figure 7B).

PIICP and COMP release was increased after compression in the affected compartment (*p* = 0.013 and *p* = 0.018, respectively); the same trend, even without reaching statistical significance, was observed in the contralateral compartment and both in the anterior and posterior areas (Figures 7C,D).

Finally, CS release was again stimulated by compression, both in the affected and in the contralateral compartments (*p* = 0.006 affected; *p* = 0.006 contralateral). The trend was the same if considering sample position, with a statistically significant increase in the posterior affected compartment and in the anterior contralateral compartment (Figure 7E).

## 4 Discussion

In the present study, human cartilage explants from mono-compartmental knee OA femoral condyles were individually analysed and subjected *in vitro* to a loading regimen simulating the average knee loading during normal walking. Cartilage areas were separated according to the disease score and to the anatomical position, and were analysed for chondrocyte gene expression and for release of factors from the ECM. We observed an anti-inflammatory effect of compression inducing a significant downmodulation of IL-6 and IL8 levels correlated to the anatomical regions, but not to the OA score. Moreover, ADAMTS5, PIICP, COMP, and CS were upregulated by compression, whereas COL-2CAV was downmodulated, all in relationship to the anatomical position and to the OA degree.

The results of this study provide further evidence on the relationship between the mechanics of the most common activity of daily living, i.e., walking, and the OA degree with markers expression in patients with mono-compartmental knee OA. A recently proposed original investigation methodology in mechanotransduction (Caravaggi et al., 2021) was here used for the *in vitro* testing of human explanted knee cartilage tissue, based on physiological biomechanical data, and according to cartilage scoring and anatomical position.

The analysis of gene expression and protein production data obtained from *in vitro* experiments was based on the anatomical features of mono-compartmental knee OA, this being caused by asymmetric overloading due to the misalignment between femoral and tibial axes. By complying with this macro anatomical difference, the comparison between the affected and contralateral compartment allowed to focus the analysis on the changes due to mechanical load and cartilage degeneration. The reliability of the sample partition in regions with different OA degrees before compression was confirmed by the positive correlation between macroscopic and histologic microscopic scores. Moreover, although high interindividual variability was observed for gene expression, this was demonstrated to be affected by the varus/valgus knee alignment, as highlighted by differences in gene expression and soluble factor release after loading. Indeed, condyle areas with higher disease score differently responded to compression compared to non-affected compartments. Conversely, no significant differences were found between the disease degree and the anatomical regions. Response to loading was investigated in basal conditions and after IL-1β treatment.

In terms of culture supernatants, the level of soluble factor release was found significantly different between the varus and valgus cartilage samples, although some peptides were undetectable. This is somehow in agreement with what reported by Erhart-Hledik et al. (Erhart-Hledik et al., 2015), that investigated the associations between cartilage thickness and knee kinetics, albeit no correlation with gene expression was performed.

Collagen 2 and Aggrecan, two of the main ECM components, and SOX9, a transcription factor which regulates also the production of Collagen 2, were here analyzed. Collagen 2 and SOX9 did not appear to be modulated by compression or by the pro-inflammatory stimulus. These results seem to be in contrast with previous observations by our group (Dolzani et al., 2019), due to differences in compression conditions. In fact, in the present experimental protocol a significantly shorter duration than what performed in the previous study was chosen to replicate the mechanical stimuli the cartilage is subjected to during a moderate walk. Furthermore, the great interindividual variability in the basal expression of these genes (Supplementary Table S2) hindered the detection of marker modulation. Some differences in Collagen 2 and aggrecan expression were observed between affected and contralateral compartments, thus confirming the importance of pooling and analyzing samples with respect to the anatomical region.

As for the analysis of the innate immune response and inflammation, this was performed on the subset of samples stimulated with IL-1β, this allowing the pro-inflammatory response amplification and the increased possibility to detect differences due to mechanical stimulation.

A similar trend was observed for the pro-inflammatory cytokines IL-6 and IL-8, which were downmodulated by load, indicating an anti-inflammatory effect of compression. These interleukins have a pleiotropic effect, are involved in OA pathogenesis and play a central role in inflammatory processes (Kapoor et al., 2011; Koh et al., 2020). Interestingly, the effect of compression was statistically significant only in cartilage samples from the anterior contralateral, i.e., less affected, compartment. This may suggest that cartilage with a lower OA grade could benefit from loading, whereas highly degraded OA does not respond to compression. Considering possible rehabilitation protocols, specific exercises could be beneficial under mild or less severe OA. On the other side, the significant anti-inflammatory effect observed only in the anterior contralateral compartment suggested that cartilage response to mechanical loading is dependent on the original anatomical region and to the associated *in vivo* loading.

In agreement with what reported previously (Dolzani et al., 2019), we did not observe a significant effect of compression on IL-4Rα expression. IL-4 is an interleukin involved in the mechano-transduction and IL-4Rα is the main subunit of its receptor. The expression of the receptor is modulated in chondrocytes and particularly in OA cartilage (Assirelli et al., 2014). Our data could suggest that IL-4Rα pathway activity is substituted by mechanical compression and therefore could be used less frequently in the present experimental conditions. Notably, IL-6, IL-8, and IL-4Rα expression significantly correlated with OA score, which was higher in the affected compartment.

Although C3 and CFB expressions showed a decreasing trend in the contralateral compartment samples and were correlated to OA score, these were not significantly modulated by compression. Correlation with OA score was present also for IL-6 and IL-8. Local and systemic low-grade inflammation, via innate immunity complement pathway activation, is recognized as one of the major triggers of OA (Sokolove and Lepus, 2013). In OA, innate immunity is locally stimulated by ECM debris released by cartilage degradation (Hazlehurst et al., 2014). It has also been demonstrated that COMP exerts a dual effect on the complement cascade as it can inhibit both the classical and lectin pathways and, at the same time, it can activate the complement alternative pathway (Happonen et al., 2010). To our knowledge, no previous studies investigated innate immunity or complement pathway mechanical modulation in cartilage. A trend for decreasing inflammatory markers after compression is rather clear across all present data.

We evaluated a series of soluble markers of matrix remodeling in culture supernatants. Generally, these appeared to be the most affected by compression. The collagen cleavage biomarker COL-2CAV showed a downmodulation trend by compression which was statistically significant in the contralateral samples. COL-2CAV is released as a product of ECM degradation; several studies showed that modulation of type II collagen degradative biomarkers, such as COL-2CAV, is directly associated with the increased turnover of cartilage degradation (Hunter et al., 2007; Boeth et al., 2017). This is consistent with the hypothesis that physiological compression could have a beneficial effect in reducing ECM degradation. In agreement with this hypothesis, two ECM structural components (COMP and CS) and the collagen synthesis biomarker PIICP were augmented after compression. These are constitutive components of the cartilage matrix, and are reported to steadily decrease during OA progression (López-Franco et al., 2016; Lin et al., 2020). The applied compression regimen may therefore help to enhance their production.

ADAMTS5, an ECM degrading enzyme, was augmented by compression in the contralateral compartment. This aggrecanase is the major aggrecan-degrading enzyme in OA and is regarded as a critical factor in metabolism, homeostasis, and pathological changes of joint ECM (Jiang et al., 2021). Beside its detrimental effect, ECM degradation is a tightly regulated physiological mechanism that maintains cartilage hemostasis.

Regarding the soluble factors, a recent study by Mählich et al. (Mählich et al., 2021) investigated ECM components expression in different articular sub-areas. It was found a different expression of these factors with respect to OARSI score and to the anatomical position, similarly to our findings.

The findings reported in the present study should be interpreted with respect to the limitations of the mechanical stimuli setup. In fact, the cartilage samples were compressed in unconfined spatial conditions, which are not fully representative of the *in-vivo* biomechanical situation. This may have resulted in a larger deformation of the cylindric samples in the vertical and radial direction thus affecting the biological cartilage response. Improvement of the testing setup should be sought in future investigations. As far as the dynamic loading conditions applied to the specimens, it should be highlighted that no differentiation was made with respect to the OA degree and to the patient’s body characteristics or gender. Therefore, it may be possible that some cartilage samples were subjected to slightly larger or lower stresses compared to *in vivo* conditions. On the other hand, this study aimed at evaluating how different areas responded to the same physiologic stimulus, not at applying different stimulations.

Although, it is acknowledged that physical activity might prevent chronic diseases or slow down their progression, additional investigations should be sought to provide more evidence in this respect using more proper mechanical stimuli and environment (Davis et al., 2021) also by replicating other common daily living activities.

The correlations identified in the present study can contribute to the design of patient-based conservative treatments and rehabilitation protocols. The applied frequency rate and the loading conditions suggest that a good response occurs in terms of pro-inflammatory cytokines under mechanical stimuli replicating a mild activity like walking. In addition, an interesting region-dependent variable response to loading was observed for some markers in relation to the anatomical position. This was also true in OA grading, revealing that there could be a degree of tissue degeneration beyond which physical activity could fail in stimulating tissue anabolism and counteract inflammation.

## Data Availability

The original contributions presented in the study are included in the article/Supplementary Material, further inquiries can be directed to the corresponding author.
