# Supplementary material for: Location-Dependent Human Osteoarthritis Cartilage Response to Realistic Cyclic Loading: Ex-Vivo Analysis on Different Knee Compartments
Source: Front Bioeng Biotechnol. 2022 Jun 15;10:862254. doi: 10.3389/fbioe.2022.862254 (PMC9240619; doi:10.3389/fbioe.2022.862254)
Supplement: Supplementary file 1 [file DataSheet1.docx]

**SUPPLEMENTARY**

**MATERIAL**

| **Gene** | **Primer Forward** | **Primer Reverse** |
| --- | --- | --- |
| COL2A1 | 5'-GACAATCTGGCTCCCAAC-3' | 5'-ACAGTCTTGCCCCACTTAC-3' |
| SOX9 | 5'-GAGCAGACGCACATCTC-3' | 5'-CCTGGGATTGCCCCGA-3' |
| Aggrecan | 5'-GTCTCACTGCCCAACTAC-3' | 5'-GGAACACGATGCCTTTCAC-3' |
| IL-4Rα | 5'-TGGGCGTCAGCGTTTCCTGC -3' | 5' -CTGCGGGCTGGGTTGGGAAT- 3' |
| C3 | 5'-TCAACCACAAGCTGCTACCC - 3' | 5'-CTGGCCCATGTTGACGAGTT-3’ |
| CFB | 5'-GGGGTAGAGATCAAAGGCGG -3' | 5'-TGGATTGCTCTGCACTCTGC-3’ |
| IL-6 | 5’- TAGTGAGGAACAAGCCAGAG -3' | 5’- GCGCAGAATGAGATGAGTTG-3’ |
| IL-8 | 5’- CCAAACCTTTCCACCC -3' | 5’- ACTTCTCCACAACCCT-3’ |
| GAPDH | 5’-TGGTATCGTGGAAGGACTCATGAC-3’ | 5’-ATGCCAGTGAGCTTCCCGTTCAGC-3’ |

**Supplementary Table S1 -** Primers used for Real-Time semi-quantitative PCR*.*


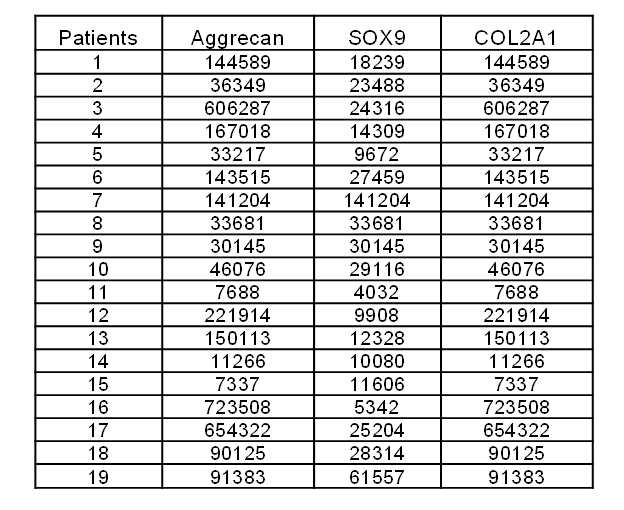


**Supplementary Table S2**. Interindividual variability in Aggrecan, SOX9 and COL2A1 gene expression. Medians of relative gene expression obtained in all samples of the same donor is shown. Values represent the number of molecules of the gene of interest/10^5^ GAPDH molecules.

|  | | | | | | |  | |  | |
| --- | --- | --- | --- | --- | --- | --- | --- | --- | --- | --- |
|  | **IL-6** | | **IL-8** | | **IL-4Rα** | | **C3** | | **CFB** | |
| **Compartment** | **Varus** | **Valgus** | **Varus** | **Valgus** | **Varus** | **Valgus** | **Varus** | **Valgus** | **Varus** | **Valgus** |
|  | 26.35 [3.55-156.46] | 8.05 [0.73-37.26] | 142.11 [11.01-1432.24] | 133.40 [26.90-579.89] | 70.50 [27.09-219.74] | 43.10  [25.81-96.31] | 50.91 [13.73-245.51] | 23.10 [9.64-61.80] | 6381.33 [4419.42-8717.15] | 4177.60 [2179.20-6250] |
|  | p=ns | | p=ns | | p=ns | | p=ns | | p=0.054 | |
| **Sample position** | **Anterior** | **Posterior** | **Anterior** | **Posterior** | **Anterior** | **Posterior** | **Anterior** | **Posterior** | **Anterior** | **Posterior** |
|  | 66.76 [13.60-555.30] | 32.27 [14.46- 2490] | 163.50 [41.13- 3037] | 154.30 [59.21- 4754] | 72.74 [31.94- 160.30] | 81.55 [41.42- 205.30] | 63.54 [20.53 – 216.60] | 47.08 [23.10- 1003] | 5954 [1897 - 9605] | 6381 [3349-7484] |
|  | p=ns | | p=ns | | p=ns | | p=ns | | p=ns | |
|  | **Medial** | **Lateral** | **Medial** | **Lateral** | **Medial** | **Lateral** | **Medial** | **Lateral** | **Medial** | **Lateral** |
|  | 52.62 [13.36- 1833] | 27.28 [12.89-155.40] | 317.30 [83.90- 2276] | 148 [26.90- 3280] | 72.99 [32.88- 147] | 75.30 [39.94-162] | 58.65 [18.18 -153.8] | 38.05 [14.95 – 294.6] | 10940.93 [6320.13-18940.10] | 4848.69 [3641.04-6456.89] |
|  | p=ns | | p=ns | | p=ns | | p=ns | | p=0.007 | |
| **Grading** | **0-1** | **3-4** | **0-1** | **3-4** | **0-1** | **3-4** | **0-1** | **3-4** | **0-1** | **3-4** |
|  | 24.75 [43.45- 1731] | 138.80 [9.71-46.19] | 93.03 [102-1923] | 387.90 [15.03-340.10] | 54.74 [46.84- 270.50] | 112.20 [27.89- 118.40] | 35.47 [19.83 – 349.60] | 78.77 [14.06 – 63.11] | 5711 [4147-8840] | 6337 [1852 - 8022] |
|  | p=0.016 | | p=0.0139 | | p=0.0362 | | p=ns | | p=ns | |

**Supplementary Table S3.** Gene expression analysis of IL-6, IL-8, IL-4Rα, C3, and CFB in relationship to disease grading, anatomical position, and varus/valgus condition (Sidak test for multiple comparisons). Reported values correspond to medians, 25th-75th percentiles (under brackets).

|  | | | | | | |  | |  | |
| --- | --- | --- | --- | --- | --- | --- | --- | --- | --- | --- |
|  | **ADAMTS5** | | **CS** | | **COMP** | | PIICP | | **Col-2CAV** | |
| **Compartment** | **Varus** | **Valgus** | **Varus** | **Valgus** | **Varus** | **Valgus** | **Varus** | **Valgus** | **Varus** | **Valgus** |
|  | 16.36 [4.62-51.82] | 4.57  [0-13.99] | 11.75 [5.92-48.10] | 23.09  [9.04-45.55] | 0.23 [0.12-0.33] | 0.29  [0.14-0.41] | 0 [0 -0.90] | 0.22  [0-1.79] | 1.08 [0.77 -1.30] | 1.38  [1-1.83] |
|  | p=0.027 | | p=ns | | p=ns | | p=ns | | p=0.025 | |
| **Sample position** | **Anterior** | **Posterior** | **Anterior** | **Posterior** | **Anterior** | **Posterior** | **Anterior** | **Posterior** | **Anterior** | **Posterior** |
|  | 878 [0-23.84] | 6.88  [1.60-27.80] | 18.01  [6.55-36.38] | 13.15  [7.06-50.90] | 0.26  [0.14-0.34] | 0.18  [0.08-0.33] | 0 [0 -1.05] | 0  [0-0.9] | 1.19 [0.87 -1.53] | 1.04  [0.77-1.32] |
|  | p=ns | | p=ns | | p=ns | | p=ns | | p=ns | |
|  | **Medial** | **Lateral** | **Medial** | **Lateral** | **Medial** | **Lateral** | **Medial** | **Lateral** | **Medial** | **Lateral** |
|  | 6.11[0-33.20] | 12.69  [2.49-31.72] | 17.68  [9.03-50.18] | 12.24  [5.64-46.8] | 0.29  [0.15-0.49] | 0.24  [0.09-0.32] | 0.65  [0-2.32] | 0  [0-0.22] | 1.25 [0.95-1.78] | 0.96  [0.75-1.3] |
|  | p=ns | | p=ns | | p=ns | | p=0.009 | | p=0.011 | |
| **Grading** | **0-1** | **3-4** | **0-1** | **3-4** | **0-1** | **3-4** | **0-1** | **3-4** | **0-1** | **3-4** |
|  | 15.45  [5.9-41.7] | 22.5  [5.6-99.4] | 9.08 [4.98-25.73] | 27.47 [11.5-64.69] | 0.22 [0.09-0.33] | 0.26 [0.15-0.37] | 0 [0-1.36] | 0 [0-0.79] | 0.95 [0.77-1.36] | 1.25 [0,90-4,55] |
|  | p=ns | | p=ns | | p=ns | | p=ns | | p=ns | |

**Supplementary Table S4.** Soluble factors release in relationship to disease grading, anatomical position and varus/valgus condition (Sidak test for multiple comparisons). Reported values correspond to medians, 25^th^-75^th^ percentiles (under brackets).
